# Drying: A Practical Technology for Blueberries (*Vaccinium corymbosum* L.)—Processes and their Effects on Selected Health-Promoting Properties

**DOI:** 10.3390/antiox13121554

**Published:** 2024-12-18

**Authors:** Elsa Uribe, Antonio Vega-Galvez, Alexis Pasten, Kong Shun Ah-Hen, Nicol Mejias, Lorena Sepúlveda, Jacqueline Poblete, Luis S. Gomez-Perez

**Affiliations:** 1 Food Engineering Department, Universidad de La Serena, Av. Raúl Bitrán 1305, La Serena 1700000, Chile; muribe@userena.cl (E.U.); afpasten@userena.cl (A.P.); susana.mejias@userena.cl (N.M.); lorena.sepulvedac@userena.cl (L.S.); j.pobletegalleguillos@gmail.com (J.P.); 2 Instituto Multidisciplinario de Investigación y Postgrado, Universidad de La Serena, Av. Raúl Bitrán 1305, La Serena 1700000, Chile; 3 Facultad de Ciencias Agrarias y Alimentarias, Instituto de Ciencia y Tecnología de los Alimentos, Universidad Austral de Chile, Valdivia 5090000, Chile; kshun@uach.cl; 4 Centro de Biología Integrativa, Facultad de Ciencias, Universidad Mayor, Camino La Pirámide 5750, Santiago 8580745, Chile; luis.gomezpe@umayor.cl; 5 Escuela Nutrición y Dietética, Facultad de Medicina y Ciencias de la Salud, Universidad Mayor, Camino La Pirámide 5750, Santiago 8580745, Chile

**Keywords:** antioxidant activity, antiproliferative, biocompounds, drying

## Abstract

The global dried blueberry market is steadily growing, driven by the creation of innovative blueberry-based products. This trend presents an opportunity to explore a previously untapped segment of the blueberry market in Chile. In this study, a comprehensive assessment of four drying techniques (hot-air drying [HAD], vacuum drying [VD], infrared drying [IRD], and freeze-drying [FD]) was conducted to determine best operating conditions and preserve the health-promoting properties of blueberries. Drying kinetics, proximate composition, color, anthocyanin content, individual phenols, and antioxidant, antiproliferative, and antidiabetic potential of blueberries were evaluated. VD showed the highest drying rates, reaching equilibrium moisture more rapidly (*D_eff_* value of 3.44 × 10^−10^ m^2^/s). Drying caused an increase in lipid content but a decrease in protein content. The color parameter L* increased in all dried samples, and C^*^ reflected color intensification. FD best retained anthocyanin content, which decreased significantly in the other drying processes. Chlorogenic acid and rutin predominated in HAD, IRD, and FD samples. The antioxidant potential in ORAC assays increased for all drying methods but decreased in DPPH assays. Blueberry extracts from FD and HAD exhibited the greatest antiproliferative effect against A549 and H1299 cell lines, respectively. HAD showed the best inhibitory effect on α-glucosidase, with an IC50 value of 0.276 mg/mL, similar to acarbose (IC50 = 0.253 mg/mL). Given the significant retention of health-promoting properties and bioactive compounds in HAD-dried samples, this method is advisable as a sustainable option for drying blueberries in Chile.

## 1. Introduction

Blueberries (*Vaccinium corymbosum* L.) are a crop of major economic importance with a rapidly increasing market size. The worldwide production of blueberries increased more than threefold from 2012 to 2022, from 489,965.1 t to 1,228,596.0 t. In South America, the production of blueberries increased more than fivefold in the same period, from 78,560.0 t to 415,096.2 t. The production in Chile achieved 122,512.15 t, representing an increase in gross production value of 57%, corresponding to 303,165,000 INT$ [1,2]. Blueberries are known for their numerous beneficial health properties, mostly associated with the high polyphenol content of the berries. Phytochemicals, such as anthocyanins, flavonoids, phenolic acids, and stilbenes, have biological activities that reduce inflammation, prevent cancer, enhance cognitive function, lower blood pressure, and improve insulin sensitivity, contributing to the potential of blueberry extracts for therapeutic use [3,4,5].

According to the literature, the phenolic compounds that predominate in blueberries are chlorogenic acid (5-caffeoylquinic acid), anthocyanins, and proanthocyanidins. Other minor phenolics found in blueberries are flavonoids and hydroxycinnamic acids [6,7]. Blueberry anthocyanins have been extensively studied and have been shown to effectively quench reactive oxygen species. In vitro studies have revealed that anthocyanin extracts from the blueberry cultivar Polaris promoted cellular activity of HepG2 cells, including the levels of superoxide dismutase, catalase, and inhibition of malondialdehyde [8]. Blueberry anthocyanins have also been reported to improve vision by reducing diabetic retinopathy, age-related macular degeneration, and night vision [9]. Therefore, habitual consumption of blueberries is expected to regulate postprandial glucose fluctuation in the body through improvement of the glycemic index of meals [10,11]. In fact, the predominant chlorogenic acid in blueberries has also been reported to contribute to regulating blood glucose, improving insulin resistance, and reducing the risk of type 2 diabetes by an estimated dietary intake of 5 to 1000 mg/d [12].

Nevertheless, after harvesting, the native polyphenols in blueberries are susceptible to oxidative reactions due to their high water content, which can diminish their beneficial health properties. Therefore, a unit operation such as drying is required to remove water and retain these beneficial properties. Before selecting a drying technique, several factors must be considered. These include physicochemical characteristics such as composition, cellular structure, hygroscopic nature, heat susceptibility or tolerance, as well as the desired properties of the dried products, along with economic and environmental considerations. The synchronization of all these heterogeneous factors in a drying process is a complex matter the is hard to fulfill and requires continuous research initiatives. Recently, numerous novel drying methods have been applied to produce dried blueberries, each varying in the heat transfer mechanism (conduction, convection, or radiation). Kapoor and Feng [13] showed, for example, that dried blueberries prepared by InfriDri^®^ Process (IFD) possessed higher total phenolic content (TPC) and free radical scavenging activity (DPPH) values, though these differences were not statistically significant (*p* > 0.05) compared to those prepared via freeze-drying (FD). Liu et al. [14] found that pulsed vacuum drying (PVD) enhances the drying of blueberries, resulting in higher TPC and total monomeric anthocyanins (TMA) than those obtained by hot-air drying (HAD). Later, Liu et al. [15] applied far-infrared radiation heating-assisted pulsed vacuum drying (FIR-PVD) to process blueberries and also noted that TPC, TMA, and antioxidant values of the dried product were higher than those of HAD products. Moreover, several authors have considered different novel pretreatments to accelerate the drying rate of blueberries due to the external waxy skin of the fruit [16,17]. While these novel drying technologies and pretreatment methods have demonstrated promising results in laboratory settings, their scalability to industrial applications in Chile still remains a significant challenge.

Chile is the main exporter of frozen blueberries and the second largest exporter of fresh blueberries in the southern hemisphere. In the current 2023/2024 season, Chile exported 32,248 tons of frozen blueberries and 86,264 tons of fresh blueberries, with the United States and Canada as the major markets, followed by Europe and Asia [2]. However, a recent market study valued the global dried blueberries market at USD 5.79 billion in 2023, projecting it to reach USD 9.59 billion by 2031 [18]. This presents an opportunity to explore a previously untapped segment of the blueberry market in Chile. Drying processes are necessary in the production of extracts and ingredients from blueberries; so the assessment of the effects of drying is determinative for the quality maintenance of the dried berries. The worldwide market for blueberry ingredients is expected to have a compound annual growth rate (CAGR) of 7.3% from 2023 to 2030, while the global blueberry extract market is projected to grow at a CAGR of 10.09% over the period of 2021–2026 [3].

Therefore, this study aims to determine how various drying techniques, including hot-air drying, vacuum drying, infrared drying, and freeze-drying, affect the properties of blueberries essential for the production of extracts and ingredients. Through the evaluation of drying kinetics, proximate composition, color characteristics, phenolic and anthocyanin contents, as well as antioxidant, antiproliferative, and antidiabetic activities, the drying impact on the blueberries was assessed, aiming to identify an optimal drying technique that best preserves the bioactive properties and health benefits of the dried product.

## 2. Materials and Methods

### 2.1. Solvents and Reagents

All reagents and solvents used were of analytical grade and were obtained from SIGMA Aldrich (St. Louis, MO, USA): methanol (MeOH), hydrochloric acid (HCl), sodium hydroxide (NaOH), Folin–Ciocalteu reagent, sodium carbonate (Na_2_CO_3_), formic acid, acetonitrile (C_2_H_3_N), 6-Hydroxy-2,5,7,8-tetramethylchoman-2-carboxylic acid (Trolox), 2,2-diphenyl-1-picrylhydrazyl (DPPH), 2,2-azobis(2-amidinopropane) dihydrochloride (AAPH), gallic acid, quercetin, propidium iodide, α-glucosidase (*S. cerevisiae*; Sigma G50034), and nitrophenyl α-D-glucopyranoside (Sigma N1377). Rohapect 10-L (Dimerco Commercial S.A., Santiago, Chile), F-12K medium (2 mM L-glutamine and 1500 mg/L NaHCO_3_) (Cytiva, Marlborough, MA, USA), RPMI 1640 medium (Cytiva, Marlborough, MA, USA), fetal bovine serum (FBS) (Hyclone, Logan, UT, USA), penicillin and streptomycin (Corning^®^, Corning, NY, USA).

### 2.2. Sample Preparation for Drying Processes

Fresh blueberries (*Vaccinium corymbosum* L.) were acquired from an established trade (Hortifrut Chile S.A., Santiago, Chile) in Chile to obtain a product complying with quality criteria in term of freshness, wholesomeness, color, and homogeneity, and were stored after reception at 4 °C for 24 h. Before the different drying processes, the blueberries were subjected to an enzymatic treatment with pectinase at 1% as reported by Vega-Gálvez et al. [19], with some modifications. The enzyme preparation Rohapect 10-L at 1% was carried out in a stirring water bath (N-Biotek, NB-302, Bucheon-si, Gyeonggi-do, Republic of Korea) at 50 °C for 30 min. The fresh pretreated blueberries were then left to drain to remove surface moisture. One batch was used as a control sample in this study, while the other batches were subjected to the four drying methods.

### 2.3. Drying Processes and Effective Diffusion Coefficient

Thirty grams of enzymatic-pretreated blueberries were placed in 14.0 × 8.0 cm baskets and subjected to four different drying methods: vacuum drying (VD), hot-air drying (HAD), infrared-radiation drying (IRD), and freeze-drying (FD). All methods except FD involved a drying temperature of 70 °C. The HAD process was carried out at a constant airspeed of 1.5 m/s, where the air flowed vertically through the blueberries during the entire drying time. Blueberries were vacuum-dried using a vacuum dryer (Memmert VO 400, Schwabach, Germany) connected to a vacuum pump (Buchi, V-100, Flawil, Switzerland) with an ultimate pressure of 10 kPa. For the IRD method, the system consisted of a combination of two 175-watt IR incandescent lamps mounted inside the oven. The blueberries were placed under the IR lamps with a distance of 22 cm between the IR source and the samples. In the case of FD, the blueberries were first frozen at −80 °C for 24 h and then dried in a freeze-dryer (Friologic, Given one 5K, Santiago, Chile), using a drying program consisting of primary drying from −20 to 20 °C with the condenser temperature adjusted to −55 °C. Secondary drying occurred at 25 °C and a vacuum of 0.027 kPa. The berry weight loss was monitored by means of a digital balance (SP402, Ohaus, Parsippany, NJ, USA) with an accuracy of ±0.01 g and registered every 30 min in VD, HAD, and IRD, and every 120 min in FD until constant weight (equilibrium condition) was reached. Each drying experiment was carried out in triplicate.

The moisture ratio (MR) of blueberry samples during drying was calculated using the following formula:

(1) $$MR=\frac{M_{t}-M_{e}}{M_{0}-M_{e}}\sim \frac{M_{t}}{M_{0}}$$

where M_0_ represents the initial moisture content (kg water/kg d.m.), M_t_ represents the moisture content at the sampling point (kg water/kg d.m.), and M_e_ represents the equilibrium moisture content (kg water/kg d.m.). For longer drying times, the value of M_e_ is small compared to M_t_ or M_0_. Therefore, M_e_ can be considered zero [20].

The drying rate (DR) was calculated using the formula shown in Equation (2):

(2) $$DR=\frac{∆M}{∆t}=\frac{M_{t}-M_{t+∆t}}{∆t}$$

where M_t_ is the moisture content at a time t, M_t+Δt_ is moisture content at time t + Δt (kg water/kg d.m.), and Δt is the time difference (min) [21].

The mass diffusion coefficient (*D_eff_*) was determined using Fick’s second law for a spherical geometry (Equation (3)), assuming that moisture migration occurs by diffusion, temperature and diffusion coefficients remain constant, and negligible shrinkage occurs during the drying process [22].

(3) $$MR=\frac{6}{\pi^{2}}exp\left[-\frac{D_{eff}\pi^{2}t}{r^{2}}\right]$$

where *r* (m) is the mean radius of the blueberries, and *t* (s) is the drying time.

### 2.4. Proximate Composition

The proximate analysis was used to determine the chemical composition of blueberries by assessing their moisture content (sample kept in an oven overnight at 105 °C), lipids (analyzed gravimetrically after Soxhlet extraction), ash (by muffle incineration at 550 °C), protein (Kjeldahl method), fiber (estimated by acid/alkaline hydrolysis of insoluble residues), and carbohydrate content (by difference). The detailed procedures to determine all of these parameters are described by AOAC (Association of Official Analytical Chemists) [23] methodologies. Water activity was also evaluated in all samples. All measurements were performed in triplicate.

### 2.5. Color Change

The color change among the control and the four types of dried blueberries was determined using a Hunter Lab color measurement instrument (MiniScan XE-Plus, Reston, VA, USA) under an illuminant D65 and a 10° observation angle. Ten grams of powdered sample was placed in a 9 cm diameter and 10 mm deep plastic Petri dish, and the surface was covered with a dark background before color measurement to avoid diffraction errors. Three readings were taken from beneath the same sample, and the dish was rotated ~120° after each reading. Six replicate measurements were performed, and the results were averaged. The L*-value indicated the level from light to dark, the a*-value indicated the range from red to green, and the b*-value indicated the range from yellow to blue. The total color change (Δ*E*), chroma (C*), and hue angle (h*) were calculated for both control and dried samples using Equations (4), (5), and (6), respectively [16]:

(4) $$\Delta E=\sqrt{{\left(L_{0}^{*}-L^{*}\right)}^{2}+{\left(a_{0}^{*}-a^{*}\right)}^{2}+{\left(b_{0}^{*}-b^{*}\right)}^{2}}$$

(5) $$C^{*}=\sqrt{a^{*2}+b^{*2}}$$

(6) $$h^{*}={\;tan}^{-1}\left(\frac{b^{*}}{a^{*}}\right)$$

### 2.6. Extraction of Compounds

To prepare the extracts, control and dried blueberries were weighed (in grams) in centrifuge tubes and extracted with 20 mL of methanol/water (50:50, *v*/*v*), agitated on an orbital shaker (OS-100, HiLab, Bahasa, Indonesia) at 20 °C and 200 rpm for 1 h. The tubes were centrifuged (Eppendorf, 5804 R, Hamburg, Germany) at 10,000× *g* for 15 min at 5 °C, and the supernatants were recovered. Then, 20 mL of acetone/water (70:30, *v*/*v*) was added to the residue, agitated for 1 h, and thereafter centrifuged according to de Souza et al. [24]. Both supernatants were collected, combined, and concentrated at 40 °C on a rotary vacuum evaporator (Büchi, R-210, Flawil, Switzerland) until the solvents were removed completely. The resultant aqueous extract was lyophilized (solid extract) and then stored at −20 °C until further testing.

### 2.7. Total Anthocyanin Content (TAC)

The pH differential assay, as suggested by An et al. [22], was used to determine the total anthocyanin content (TAC) of blueberries. The solid extracts obtained previously were transferred into a volumetric flask, and methanol/water (80:20, *v*/*v*) was added to adjust the volume to 5 mL. Then, one aliquot of extract was diluted with a buffer solution of either pH 1.0 or pH 4.5, and stored in dark for 15 min. The absorbance was read at 510 nm and 700 nm for each of the buffers. The TAC was expressed as cyanidin-3-glucoside equivalent (mg C3G/100 g d.m.). A molar absorptivity of 26,900 was used for C3G (molecular weight 449.2).

### 2.8. Identification and Quantification of Phenolic Compounds

The phenolic compounds in the blueberry samples were identified and quantified using high-performance liquid chromatography with a diode array detector (HPLC-DAD). The HPLC system (Agilent 1200 series, Santa Clara, CA, USA) was equipped with a Kromasil 100-5C18 column (250 × 4.6 mm; Eka Chemical, Bohus, Sweden) with a spherical particle size of 5 μm and run at 25 °C with a flow rate of 0.7 mL/min. Briefly, the solid extract was weighed accurately (20 mg) and mixed thoroughly with 1 mL of methanol: formic acid (99:1), and then filtered through a 0.45 μm membrane filter. The injection volume was 10 μL, and the mobile phase consisted of 0.1% formic acid (A) at pH 2.6 and 100% acetonitrile (B). The elution gradient was initially set at 87% A and 13% B. From 0–16 min, it changed to 45% A and 55% B, 16–23 min to 60% A and 40% B, 23–25 min to 87% A and 13% B, and at 25–30 min, it returned to the initial conditions (87% A and 13% B). The absorption spectra for the main peaks were read at wavelengths of 280, 310, and 370 nm [25].

### 2.9. Antioxidant Potential (DPPH and ORAC Assays)

The 2,2-diphenyl-1-picrylhydrazyl (DPPH) assay was performed following the methodology of Grajeda-Iglesias et al. [26], with minor modifications. First, 20 μL of the reconstituted solid extract in 5 mL of methanol/water (80:20, *v*/*v*) was mixed with 180 μL of DPPH (120 μM) in a transparent 96-well microplate. The microplates were incubated for 30 min while protected from light. Absorbance was then read in a multiplate reader (Perkin-Elmer, Victor TM X3, Hamburg, Germany) at 517 nm. Results were expressed as μmol Trolox Equivalent (TE)/g d.m. (y = −0.0014x + 0.6844; R^2^ = 0.9914). To conduct the Oxygen Radical Absorbance Capacity (ORAC) assay [27], 40 μL of reconstituted extracts were diluted in an assay buffer solution (75 mM phosphate buffer solution, pH 7.4) and combined with a fluorescein sodium salt solution (200 μL, 100 nM). The reaction mixture was then added to black 96-well plates and incubated at 37 °C for 20 min, followed by the addition of AAPH solution (35 μL, 0.36 M). Fluorescence intensity was measured using a fluorescence plate reader at 37 °C (excitation: 485 nm; emission: 535 nm) every minute until the reading decreased. A calibration curve was established with Trolox as the standard, with concentrations ranging from 5 to 250 μM (y = −0.0012x + 0.5901; R^2^ = 0.9808). Results were expressed as μmol TE/g d.m.

### 2.10. Antiproliferative Potential (Cell Viability Assay)

Two lung cancer cell lines, H1299 and A549, were used to determine antiproliferative activity. Cells were grown in RPMI and F12-K media, respectively, supplemented with 10% fetal bovine serum and 1% penicillin-streptomycin, in a CO_2_ incubator (Memmert, INCO153med, Schwabach, Germany) at 37 °C, 97% relative humidity, and 0.5% CO_2_. Cells were seeded in a black 96-well plate (5000 cells per well) and allowed to set for 24 h. After discarding the culture medium, different concentrations of solid extracts reconstituted in RPMI medium (50 to 3200 µg/mL) were added and incubated for 48 h. Finally, propidium iodide was added, and after 10 min in the incubator, fluorescence was measured with a microplate reader (TECAN, Instrument INFINITE 200 PRO, Männedorf, Switzerland) at λ_ex_ = 535 nm and λ_em_ = 617 nm. Results were expressed as a percentage of cell viability ± SEM. The IC50 was determined by nonlinear regression with RStudio.

### 2.11. Antidiabetic Potential (α-Glucosidase Inhibition)

The α-glucosidase inhibition assay of the blueberry extracts was performed according to a previously described method, with modifications [28]. Briefly, the solid extract was accurately weighed (20 mg) and mixed thoroughly with 1 mL of methanol/water (80:20, *v*/*v*). The α-glucosidase from yeast was dissolved in phosphate buffer (0.1 M), and 4-nitrophenyl-α-D-glucopyranoside (pNPG) was selected as a substrate. Then, 50 μL of different concentrations (0.010–20.0 mg/mL) of extracts was mixed with 100 μL of 0.5 U/mL α-glucosidase solution and pre-incubated at 25 °C for 10 min. After the pre-incubation, 50 μL of substrate (5 mmol/L) was added to the mixture to start the reaction. Absorbance readings were recorded at 405 nm using a multiplate reader every 30 s for 10 min at 25 °C. In place of the blueberry extracts, phosphate buffer and acarbose were used as a blank group and positive control, respectively. The rate of inhibition (%) on α-glucosidase by the inhibitor (blueberries extracts or acarbose) was calculated as follows:

(7) $$\%\;inhibition=\left(\left[\frac{{∆A}_{405}^{blank}-{∆A}_{405}^{inhibitor}}{{∆A}_{405}^{blank}}\right]\right)\times 100$$

where ${∆A}_{405}^{blank}$ represents the absorbance of the blank at the beginning and end of incubation, whereas ${∆A}_{405}^{inhibitor}$ represents the absorbance of the inhibitor at the beginning and end of incubation. A substrate was present in all these groups. All assays were carried out in triplicate.

### 2.12. Statistical Analysis

Analyses in triplicate (*n* = 3) were performed for proximate composition, anthocyanin content, individual phenols, antioxidant, antiproliferative, and antidiabetic potential of blueberries, whereas six measurements were carried out for the color parameters (*n* = 6). The data are presented as mean ± standard deviation (SD). Only in the antiproliferative analysis, data are presented as mean ± standard error of the mean (SEM). All data obtained from the determinations were analyzed statistically using Statgraphics Centurion Version 18.1.12 (Statgraphics Technologies, Inc., The Plains, VA, USA). The One-Factor ANOVA and Duncan’s multiple range test were used for comparison of the experimental results. A *p*-value less than 0.05 (*p* < 0.05) was considered statistically significant. Python version 3.9.4 (Python Software Foundation, Beaverton, OR, USA) was used to create heat map plots that displayed the Pearson correlation between the variables (Figure S1).

## 3. Results and Discussion

### 3.1. Drying Curves

**Figure 1:**
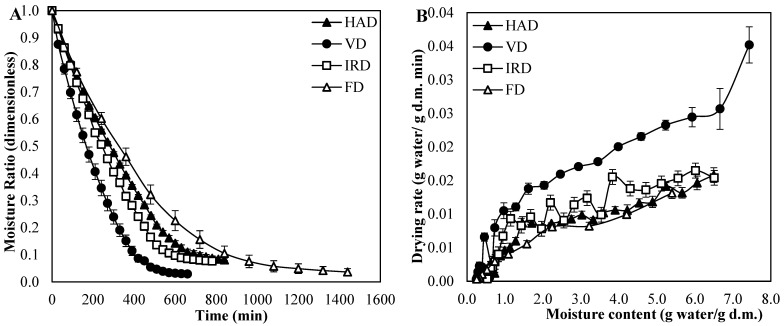
(**A**) Effects of drying methods on the moisture ratio (MR) versus drying time curve of blueberries. (**B**) Drying rate (DR) versus drying time curve for blueberries with different drying methods. Values are the means of triplicate analyses (*n* = 3), and error bars are the standard deviation. Hot-air drying (HAD), vacuum drying (VD), infrared drying (IRD), and freeze-drying (FD).

The moisture ratio (MR) and drying rate (DR) of blueberries subjected to four different drying conditions are shown in Figure 1.

Figure 1A shows that the MR of blueberries decreased continuously over the process time. The drying time significantly varied according to different temperature regimes and drying conditions employed by each method. The total drying time required for reaching an asymptotic curve was 660, 780, 840, and 1440 min for blueberries dried by VD, IRD, HAD, and FD, respectively. As a result, VD reduced drying time by 54.2%, 21.4%, and 18.2%, compared to FD, HAD, and IRD, respectively. Interestingly, the dried sample obtained from VD showed the lowest MR value, followed by FD. This phenomenon might be due to the boiling point of water decreasing when the chamber pressure decreased from 101.3 to 10 kPa. Therefore, the temperature of the blueberries exceeded the boiling point, leading to intense water evaporation and a sharp drop in the fruit’s temperature [15]. Likewise, extreme vacuum conditions during the FD process facilitated sublimation of ice and the generation of tiny bubbles in the fruit, enhancing the diffusion and removal of water [16]. In contrast, during HAD and IRD, the blueberry temperature rose quickly and reached a steady state in a short time, which caused rapid surface drying and hardening, inhibiting water diffusion [15,21].

In addition, as depicted in Figure 1B, no clear constant drying rate period was observed in any of the curves, likely due to a rapid removal of non-bound water present on the fruit’s surface, which led to a negligible constant rate period [21,29]. The same behavior was observed by other authors during drying of blueberries subjected to different pretreatment and drying methods [21,29,30,31,32], agreeing that diffusion is the predominant mechanism during the drying of this fruit [33]. In the case of IRD and HAD, the berries tended to greater shrinkage during the drying process so that the internal resistance to the diffusion of water molecules increased with structural collapse, while during VD, the mass transfer occurred in an inflated space with high porosity, leading to a higher diffusion rate [34]. Moreover, once the water molecules reached the fruit surface, the greater impulsive force in the case of VD contributed to a further increase in the drying rate [14]. In the case of freeze-drying, the mass transfer occurred by sublimation of ice crystals, which is a much slower process. These observations are supported by the determined value for the effective diffusion coefficients (*D_eff_*), with VD achieving a value of 3.44 × 10^−10^ m^2^/s, which is more than twice the value achieved in FD (1.07 × 10^−10^ m^2^/s), HAD (1.40 × 10^−10^ m^2^/s), and IRD (1.61 × 10^−10^ m^2^/s) processes. These values were within the range of 10^−11^–10^−8^ reported in previous investigations for dried blueberries subjected to different drying conditions [16,19,22,29,30,35,36,37,38].

Nonetheless, further investigations are required to evaluate the energy efficiency of the aforementioned drying systems for large-scale applications in the food industry, as well as to gain a clearer understanding of the economic feasibility of drying blueberries in Chile.

### 3.2. Determination of Proximate Composition Analysis and Water Activity

Table 1 presents the values for moisture, lipids, ash, crude protein, crude fiber, total carbohydrates, and water activity (a_w_) of dried blueberry samples using four different methods.

Fresh blueberries, pre-treated enzymatically, were used as a control. A similar proximate composition analysis has been revealed in fresh blueberries by other authors [39,40,41,42].

After drying, the moisture content of the samples was homogeneous, although FD-dried samples showed a slightly lower moisture level. This indicates that the enzymatic pre-treatment promoted water removal more effectively from the blueberries through sublimation. The dried blueberries presented significantly higher (*p* < 0.05) lipid content than the fresh control (0.82 g/100 g d.m). Grinding the dried sample played a significant role in increasing the lipid content, as fatty acids may have been slightly released from the small particles [43]. Ash content also slightly increased in all the dried samples due to there being less moisture [16]. In contrast, the decreasing trend observed in the protein content of dried samples could be attributed to nonenzymatic degradation. This process occurs when reducing sugars interact with certain amino acids, forming indigestible complexes that decrease both the quality and quantity of food proteins [44]. Likewise, the fiber content decreased as the blueberry matrix dried, with VD and FD showing the lowest values. This trend may result from the partial degradation of pectin or other cell wall fibers, such as cellulose and hemicelluloses, into simpler carbohydrates, which may be induced by certain enzymes that were protected from stresses from a vacuum environment [45]. This was further confirmed by the slight increase in total carbohydrates observed in the VD and FD samples compared to the other ones. Finally, all samples exhibited similar and safe a_w_ values due to the pectinase activity, which breaks down the cell walls of blueberries, thereby facilitating moisture extraction [16].

### 3.3. Color Measurement

The CIELAB system was used to measure the color values in the control and dried blueberries by four different methods (Table 2).

The L*, a*, and b* values of fresh blueberries are 6.11, 11.59, and 0.86, respectively. In the literature, several authors, including Chen et al. [46], Akcicek et al. [33], Bei et al. [47], and Díaz-Álvarez et al. [16], have reported very different L* values, ranging from 23.54 to 35.31, from those found in our study. It is likely that the enzymatic pretreatment we applied to the fresh blueberries caused a loss of their natural brightness. After drying, the L* value increased significantly in all samples (*p* < 0.05). The FD sample showed the highest L* value (26.45), suggesting that blueberries dried by FD were considerable lighter in color than the other methods. VD, HAD, and IRD samples instead showed no significant differences (*p* > 0.05) in the L* value. Apart from the fact that non-enzymatic browning reactions do not occur in samples dried by FD, it is possible that the white waxy layer on the outer surface of the blueberry fruit increased, resulting in a brighter appearance [33]. In contrast, thermal drying degrades this waxy layer, which could explain the limited increase in the L* value in dried samples with VD, HAD, and IRD. As expected, the fresh sample and the FD sample had adjacent a* values, indicating that the red color of the FD-dried sample was closest to that of fresh blueberries. Instead, the lower a* values observed in the VD and IRD samples suggest that they were less red than the other samples. The C* value confirmed that samples subjected to VD and IRD turned grayish red, while FD-dried blueberries exhibited a more intense pinkish-red color. This may be attributed to the degradation of reddish anthocyanins, which can cause the samples to turn bluish brown [15]. Although h* values close to zero indicated that the blueberries retained their dark color during the drying process [48], all *ΔE* values were higher than 3, suggesting a noticeable color change [16]. As evident from Table 2, the L* value of blueberries increased after drying, particularly in FD, which may be the main reason for the higher Δ*E* [47].

### 3.4. Determination of Total Anthocyanins Content (TAC)

Since anthocyanins are responsible for the red coloration in blueberries, noticeable changes in the color a* parameter after drying are usually considered variations in the abundance of anthocyanins, as indicated by a strong positive correlation between these parameters (r = 0.924). As expected, the total anthocyanin content (TAC) of control blueberries was comparable to that of FD-dried blueberries, with values of 544.10 and 410.05 mg Cy-3-G/100 g d.m., respectively (Table 2). These values fell within the ranges reported previously by other authors [5,7,14,15,42,46]. A comparative anthocyanin content in fresh and FD blueberry puree was also reported by Chen and Martynenko et al. [17]. The high TAC in FD-dried blueberries might be due to freezing reducing water activity, which prevents water-induced nucleophilic attacks that directly cause the opening of the C-ring in anthocyanins. In turn, low temperatures prevent thermal cracking of anthocyanins [49]. In contrast, the dehydration of blueberries by VD, HAD, and IRD occurs at 70 °C, which highly favors the thermal degradation of anthocyanins (Table 2). The percentage of anthocyanin degradation in these dried blueberries ranges from 59% to 62%, showing no statistical differences (*p* > 0.05). In comparison, Zielinska and Michalska [32] reported that using combined drying methods (HAD at 90 °C and microwave vacuum drying) reduced the monomeric anthocyanin content in fresh blueberry pomace by 60%. An et al. [22] found that the anthocyanin content of blueberries after HAD at 60 °C was reduced by 67.7%. However, Liu et al. [14] observed that total monomeric anthocyanin of blueberries decreased by 61.1% and 81.0% in fresh samples subjected to pulsed vacuum drying at 65 °C and HAD at 65 °C, respectively. As noted, this occurrence has been observed in many works and is attributed to increased polyphenol oxidase activity caused by elevated temperatures [50].

While the data acquired in our study confirm that anthocyanins have low thermal stability, some authors have reported that the use of an adequate drying method could protect and even enhance anthocyanin content. For example, Zia and Alibas [7] found the anthocyanin content in naturally dried, microwave-dried (at 300 W), and combined microwave-convective-dried (at 100 W—90 °C) blueberries was 1.86, 2.58, and 2.57 times more than that in frozen fresh fruit, respectively. However, scaling them up to an industrial level in Chile remains a challenge.

### 3.5. Determination of Phenolic Profile—Identification and Quantification

**Figure 2:**
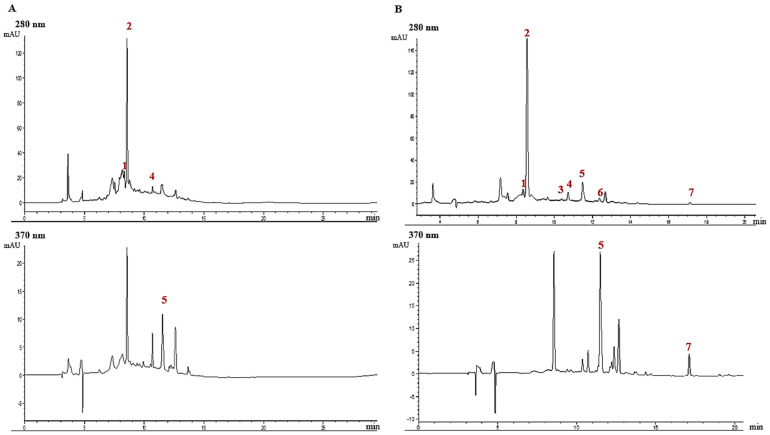
Qualitative analytical results of the phenolic profile in (**A**) control and (**B**) representative dried samples of blueberries by HPLC-DAD. **1** Protocatechuic acid; **2** Chlorogenic acid; **3** Epigallocatechin; **4** Caffeic acid; **5** Rutin; **6** Naringin; **7** Quercetin.

The qualitative analysis by HPLC-DAD of the control and dried blueberries is shown in Figure 2. Four types of phenols were identified in the control blueberries (Figure 2A), including one hydroxybenzoic acid (**peak 1**), two hydroxycinnamic acids (**peaks 2 and 4**), and one flavonoid (**peak 5**). In dried blueberries (Figure 2B), the same hydroxybenzoic and hydroxycinnamic acids were identified, along with four flavonoids (**peaks 3, 5, 6, and 7**).

The quantitative data are shown in Table 3. Chlorogenic acid was the predominant phenolic acid in the control sample, constituting 76.0% of the total compound quantities found in control blueberries. Meanwhile, minor amounts of caffeic acid, protocatechuic acid, and rutin constitute about 24.0% (Table 3). These findings are consistent with previous studies, which also reported chlorogenic acid as the major phenolic acid in various blueberry species [51,52,53,54].

The drying process caused an increase in the concentration of all identified compounds relative to the control. The FD- and HAD-dried blueberries exhibited the highest concentrations of chlorogenic acid, being 3.40 and 3.23 times greater than the concentration in the undried fruit, respectively. Likewise, the increases in VD and IRD were 1.39 and 2.86 times greater, respectively. Rutin is the second most abundant compound found in all dried blueberries, with a content that is significantly higher (*p* < 0.05) than that of the undried product. Therefore, drying processes led to the release of phenolic compounds to a degree that is dependent on the drying methods. This occurrence has been attributed to the potential effects of both thermal and non-thermal drying methods on bound compounds in the cell walls [55]. For example, Ochmian et al. [50] found that FD blueberries had phenolic acid, flavonol, and flavan-3-ol content several times higher than that of fresh fruit, likely due to the dehydration process.

Our study revealed that while FD effectively enhances the phenolic compounds of blueberries, its high cost and scalability challenges limit its widespread use. Therefore, HAD at 70 °C may offer an optimal balance between energy efficiency, compound preservation, and functional quality for industrial applications [21].

### 3.6. Determination of Antioxidant Potential

**Figure 3:**
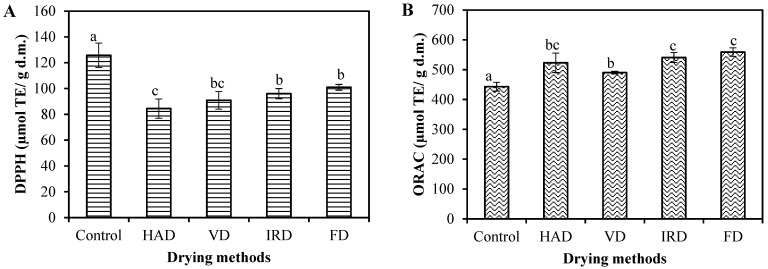
Antioxidant potential of blueberries as affected by drying methods: (**A**) 2,2-diphenyl-1-picryl-hydrazyl (DPPH) and (**B**) Oxygen Radical Absorbance Capacity (ORAC) assays. Values are the means of triplicate analyses (*n* = 3), and error bars are the standard deviation. TE: Trolox equivalents. Different alphabet letters presented on the bars indicate significant differences at *p* < 0.05 among different drying methods. Hot-air drying (HAD), vacuum drying (VD), infrared drying (IRD), and freeze-drying (FD).

The antioxidant potential of the control and dried blueberries was assessed using 2,2-diphenyl-1-picryl-hydrazyl (DPPH) and Oxygen Radical Absorbance Capacity (ORAC) assays (Figure 3).

As shown in Figure 3A, the DPPH value of undried blueberries was 125.79 μmol TE/g d.m., which is somewhat higher than the values reported in the literature [17,56]. Different drying methods, regardless of the applied conditions, caused a significant decrease (*p* < 0.05) in the antioxidant capacity of blueberries. Both FD and IRD samples showed similar results with the least drop of around 20% compared to the control sample, while HAD samples were more affected, with a loss of 33%, and the loss in VD samples was between these values. These results are consistent with the study by Akcicek et al. [33], which reported that thermal drying technologies reduce the capturing of DPPH radicals in blueberries, while FD retains them to a greater extent. Zhang et al. [57] considered high temperatures in hot-air drying to be responsible for the degradation of the main antioxidant compounds in blueberries, along with the alterations of their chemical structures that lead to shrinkage of up to 80% and increased exposure to surrounding oxygen, which favors the degradation process. Antal et al. [58] also reported better retention of DPPH antioxidant capacity in FD-dried blueberries compared to blueberries dried by combined drying methods.

On the other hand, the ORAC value in the undried blueberries was 443.01 μmol TE/g d.m. (Figure 3B), which matches those in the literature [48,56,59,60,61]. Interestingly, the ORAC measured in blueberries significantly increased after drying regardless of the drying methods (*p* < 0.05). FD and IRD samples of blueberries demonstrated the highest antioxidant activity, showing no differences between them. HAD-dried samples showed slightly lower antioxidant activity, while the lowest ORAC value was observed in the VD blueberries (Figure 3B). A study indicated that the rise in antioxidant activity after drying could be attributed to the fact that many antioxidants are naturally bound to insoluble polymers in a covalent form. When this bond is weak, the drying process can release and activate low molecular weight natural antioxidants like phenolic acids and flavonoids, thereby increasing the antioxidant activity [62]. This effect can be confirmed by the correlation analysis of such effect, since ORAC values displayed a strong positive correlation with certain identified phenolic acids and flavonoids in our study (Figure S1). For example, chlorogenic acid and rutin, which were the predominant compounds in blueberries, were significantly correlated with ORAC values (r = 0.93 and r = 0.97, respectively). Other authors reported that ORAC better reflects the antioxidant activity of anthocyanins in blueberries, as these molecules are also effective against peroxyl radicals, which would explain the higher antioxidant values obtained with the ORAC assay compared to the DPPH assay [56,63]. However, in our study, the contribution of TAC to antioxidant activity measured by the ORAC assay was negatively correlated (r = −0.49). Therefore, these assumptions need to be tested by further studies of the anthocyanin profile.

### 3.7. Determination of Antiproliferative Potential

**Figure 4:**
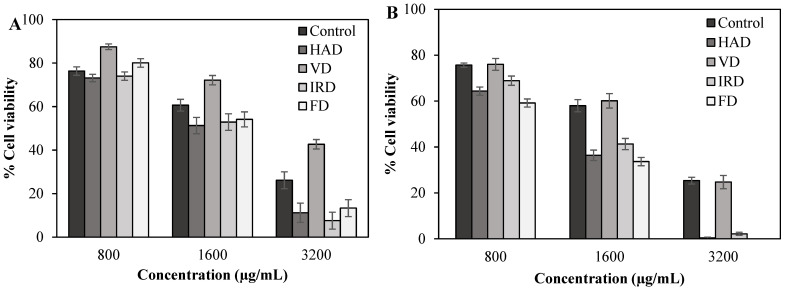
Antiproliferative effects estimated at various concentrations of extracts obtained from blueberries dried by different methods against (**A**) H1299 cell lines and (**B**) A549 cell lines. Data are expressed as mean ± SEM of three independent experiments (*n* = 3). Hot-air drying (HAD), vacuum drying (VD), infrared drying (IRD), and freeze-drying (FD).

The antiproliferative activity of control and dried blueberries was determined using a cell culture assay. Lung cancer cell lines H1299 and A549 were incubated for 48 h with blueberry extracts (50–3200 μg/mL), and cell viability was assessed by fluorescence using propidium iodide. Results are shown in Figure 4 for the three highest concentrations, showing a significant decrease in the number of viable cells in both H1299 (Figure 4A) and A549 (Figure 4B) cells, in a dose-dependent manner.

As shown in Figure 4A, the H1299 cell line was more sensitive to extracts obtained from dried blueberries by HAD, IRD, and FD, with IC50 values ranging from 1331 ± 1.22 to 1566 ± 1.53 μg/mL, with the extract obtained from HAD showing greater efficiency in reducing the number of cancerous cells by half. Likewise, Figure 4B showed that the A549 cell line had IC50 values ranging from 899 ± 1.27 to 1159 ± 1.41 μg/mL, with the extract obtained from FD being more cytotoxic toward this cell line. The higher antiproliferative activity of extracts attained from HAD, IRD, and FD may be attributed to their higher levels of chlorogenic acid and rutin, compounds that have shown a strong correlation with cancerous cell death of H1299 and A549 (Figure S1). Aligning with our findings, Yi et al. [64] reported that the phenolic acid fraction extracted from blueberries inhibited the growth of colon cancer cell lines HT-29 and Caco-2 by 50% at a concentration of approximately 1000 μg/mL. Ryu et al. [65] found that chlorogenic acid extracted from fermented blueberries inhibited HeLa cell viability by 31.13% at 200 µM, whereas catechol reduced it by 60%. Regarding blueberry processing, it has been reported that blueberries treated with high-intensity ultrasound and freeze-drying exhibited a stronger cytotoxic effect against breast cancer cells (MDA-MB-231), with rates of 52.67% and 43.41%, respectively, than those processed thermally (32.31%) [66]. Another recent study evaluated methanolic extracts obtained from dried blueberries on tongue carcinoma (HNO-97 cell line) and found an IC50 of 525.38 µg/mL [67]. Xia et al. [68] reported the cytotoxicity and antiproliferative activity of blueberry extracts, detected using HepG2 cells. The CC50 value (cytotoxic concentration 50%), a measure of the concentration of a compound that is cytotoxic to 50% of a population of cells, was observed at 80 mg/mL, suggesting that the antiproliferative activity of blueberry extracts occurred at a non-toxic concentration. While these findings support the potential of blueberry extracts in cancer treatment, further research is needed to gain a more comprehensive understanding of the mechanisms underlying their antiproliferative effects in lung cancer cells.

### 3.8. Determination of Antidiabetic Potential and Correlation Analysis

**Figure 5:**
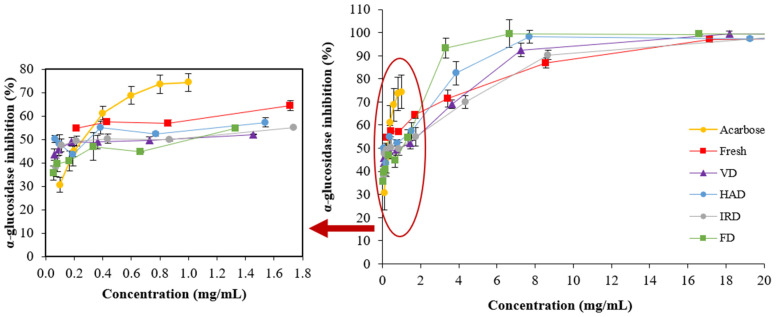
α-Glucosidase inhibitory activity estimated at various concentrations of extracts obtained from blueberries dried by different methods. Acarbose is a known anti-diabetic drug (positive control). Values are the means of triplicate analyses (*n* = 3), and error bars are the standard deviation. Hot-air drying (HAD), vacuum drying (VD), infrared drying (IRD), and freeze-drying (FD).

It is well known that controlling starch breakdown and slowing intestinal glucose absorption by inhibiting digestive enzymes, such as α-glucosidase, is considered a promising therapeutic approach for managing type II diabetes [69]. Thus, Figure 5 illustrates α-glucosidase enzyme inhibition curves exposed at multiple concentrations of acarbose (a synthetic inhibitor) or extracts obtained from dried blueberries by different drying methods (natural inhibitors).

It was obvious that both acarbose and blueberry extracts inhibited enzyme activity in a concentration-dependent manner. Specifically, a consistent increase in α-glucosidase enzyme inhibition with an increase in extract concentration in all cases is revealed. Likewise, the choice of drying method significantly affected enzyme inhibition. For example, when the concentration was 1 mg/mL (see the zoomed-in view of Figure 5), the extract obtained from dried blueberries by HAD had the best inhibitory effect on α-glucosidase, with inhibition rates of ~54.78%, followed by IRD, with inhibition rates of ~52.35%. However, both extracts were weaker than acarbose (71.91%). It is not surprising, since acarbose is a purified synthetic α-amylase inhibitor [69], whereas blueberry extracts are natural inhibitors that could be influenced by various factors, including processing.

By fitting the nonlinear curves of the α-glucosidase inhibition rate against different extract concentrations, we corroborated the best inhibitory effects on α-glucosidase for HAD, with an IC50 value of 0.276 mg/mL, followed by VD (0.328 mg/mL), IRD (0.351 mg/mL), FD (0.357 mg/mL), and fresh (0.370 mg/mL). Interestingly, these IC50 values were similar to those of the positive control acarbose (IC50 = 0.253 mg/mL). In the literature, some evidence indicates that acarbose and the anthocyanin-rich extract from blueberries followed the same trend in inhibiting α-glucosidase activity [70,71]. It has been suggested that the acylation of anthocyanins with phenolic acids considerably improves α glucosidase inhibition [70]. Zhang et al. [71] reported that the IC50 of anthocyanins and anthocyanidins from blueberries on α-glucosidase were 0.232 and 0.113 mg/mL, respectively, which fall within the ranges obtained in this study.

Although a significant correlation coefficient value (r = 0.57; *p* < 0.05) was observed between TAC and α-glucosidase inhibition at 1 mg/mL of blueberry extract concentration (Figure S1), detailed information on variations in individual anthocyanins induced by different drying methods is needed to further clarify the mechanism of α-glucosidase enzyme inhibition.

## 4. Conclusions

The findings of this study revealed that FD samples had the least impact on blueberries, maintaining a brighter color and retaining higher levels of anthocyanins, protocatechuic acid, chlorogenic acid, and rutin. At the same time, their antioxidant and antiproliferative properties improved, but FD required a longer drying time compared to the other analyzed methods. The drying time for VD was shorter than that for the other drying techniques; however, the bioactive compounds and health-promoting properties of the samples were significantly impaired (*p* < 0.05). Overall, the samples dried by HAD retained protocatechuic acid, chlorogenic acid, and rutin, comparable to FD, leading to improved antioxidant, antiproliferative, and antidiabetic properties. Considering the significant retention of health-promoting properties and bioactive compounds observed in HAD-dried blueberries in this study, HAD stands out as the most viable process for drying blueberries. Nonetheless, it is important to note that this study does not account for the calculation of the energy efficiency of drying systems. Future research should aim to evaluate the energy efficiency and successful implementation of these methods on an industrial scale in Chile while maintaining the health-promoting properties and bioactive compounds of the fruit.

## Figures and Tables

**Table 1:** Proximate composition and water activity results of control and differently dried samples of blueberries.

Parameters	Control	HAD	VD	IRD	FD
^1^ Moisture	87.46 ± 0.21 ^a^	13.68 ± 0.12 ^c^	14.10 ± 0.08 ^cb^	14.30 ± 0.01 ^b^	12.26 ± 0.69 ^d^
^2^ Lipids	0.82 ± 0.10 ^e^	1.62 ± 0.03 ^a^	1.38 ± 0.03 ^c^	1.51 ± 0.05 ^b^	1.23 ± 0.03 ^d^
^2^ Ash	1.63 ± 0.02 ^d^	1.69 ± 0.03 ^c^	1.95 ± 0.01 ^a^	1.93 ± 0.03 ^a^	1.85 ± 0.03 ^b^
^2^ Crude protein	5.34 ± 0.17 ^a^	3.70 ± 0.06 ^b^	3.40 ± 0.06 ^d^	3.63 ± 0.11 ^cb^	3.48 ± 0.11 ^de^
^2^ Crude fiber	6.94 ± 1.12 ^a^	4.19 ± 0.06 ^c^	2.57 ± 0.03 ^d^	5.15 ± 0.06 ^b^	2.43 ± 0.02 ^d^
^2^ Total carbohydrates	91.73 ± 0.54 ^c^	93.06 ± 0.03 ^ba^	93.27 ± 0.05 ^ba^	92.92 ± 0.13 ^b^	93.44 ± 0.12 ^a^
^3^ Water activity	0.9861 ± 0.0010 ^a^	0.3681 ± 0.0011 ^c^	0.3616 ± 0.0015 ^c^	0.3925 ± 0.0024 ^b^	0.3024 ± 0.0013 ^d^

^1^ Content value per 100 g fresh weight. ^2^ Content value per 100 g dry matter (d.m.). ^3^ Dimensionless. Values are expressed as mean ± standard deviation (*n* = 3). Different alphabet letters presented in the same row indicate significant differences at *p* < 0.05 among different drying methods. Hot-air drying (HAD), vacuum drying (VD), infrared drying (IRD), and freeze-drying (FD).

**Table 2:** Color parameters and total anthocyanin content (TAC) of control and differently dried samples of blueberries.

Parameters	Control	HAD	VD	IRD	FD
L*	6.11 ± 0.50 ^c^	17.17 ± 0.76 ^b^	17.48 ± 0.15 ^b^	17.52 ± 0.81 ^b^	26.45 ± 0.18 ^a^
a*	11.59 ± 0.99 ^b^	7.15 ± 0.21 ^c^	5.98 ± 0.38 ^d^	5.61 ± 0.33 ^d^	12.00 ± 0.61 ^a^
b*	0.86 ± 0.53 ^d^	1.30 ± 0.28 ^cb^	1.49 ± 0.43 ^b^	1.16 ± 0.28 ^de^	3.13 ± 0.70 ^a^
∆*E*	-	12.02 ± 0.85 ^c^	12.75 ± 0.52 ^b^	12.94 ± 1.04 ^b^	20.52 ± 0.53 ^a^
h*	0.07 ± 0.04 ^c^	0.18 ± 0.04 ^b^	0.24 ± 0.06 ^a^	0.20 ± 0.05 ^b^	0.25 ± 0.05 ^a^
C*	11.63 ± 1.01 ^b^	7.27 ± 0.19 ^c^	6.17 ± 0.43 ^dc^	5.73 ± 0.35 ^e^	12.41 ± 0.74 ^a^
^1^ TAC	544.10 ± 28.91 ^a^	212.10 ± 0.50 ^c^	225.11 ± 8.75 ^c^	209.42 ± 17.16 ^c^	410.05 ± 13.35 ^b^

Values are expressed as the mean ± standard deviation (*n* = 3). ^1^ Expressed in mg cyanidin-3-glucoside (C3G)/100 g d.m. Different alphabet letters presented in the same row indicate significant differences at *p* < 0.05 among different drying methods. Hot-air drying (HAD), vacuum drying (VD), infrared drying (IRD), and freeze-drying (FD).

**Table 3:** Quantitative analytical results of the phenolic profile in control and differently dried samples of blueberries by HPLC-DAD (mg/100 g extract).

Peak	Compounds	Control	HAD	VD	IRD	FD
1	Protocatechuic acid	31.28 ± 3.21 ^c^	50.14 ± 0.81 ^ab^	30.38 ± 1.41 ^c^	45.72 ± 2.86 ^b^	55.21 ± 0.03 ^a^
2	Chlorogenic acid	222.46 ± 4.05 ^d^	717.98 ± 9.93 ^a^	308.38 ± 9.86 ^c^	637.25 ± 20.56 ^b^	755.51 ± 24.28 ^a^
3	Epigallocatechin	ND	36.63 ± 2.21 ^a^	24.80 ± 2.03 ^c^	31.91 ± 0.64 ^b^	40.27 ± 0.34 ^a^
4	Caffeic acid	4.76 ± 0.48 ^c^	6.82 ± 0.25 ^ab^	6.34 ± 0.47 ^b^	8.00 ± 0.09 ^b^	6.47 ± 0.59 ^a^
5	Rutin	36.41 ± 1.92 ^d^	134.88 ± 1.90 ^b^	74.18 ± 1.39 ^c^	139.78 ± 3.06 ^ab^	145.48 ± 4.18 ^a^
6	Naringin	ND	24.89 ± 1.02 ^b^	19.22 ± 1.27 ^b^	26.14 ± 4.83 ^b^	36.59 ± 1.48 ^a^
7	Quercetin	ND	10.18 ± 0.08 ^b^	6.06 ± 0.20 ^c^	11.13 ± 0.80 ^a^	NQ
	TOTAL	292.65 ± 3.25 ^e^	981.51 ± 14.08 ^b^	469.36 ± 16.63 ^d^	899.93 ± 11.35 ^c^	1039.54 ± 17.66 ^a^

Values are expressed as the mean ± standard deviation (*n* = 3). ND: Not detected; NQ: Not quantifiable. Different alphabet letters presented in the same row indicate significant differences at *p* < 0.05 among different drying methods. Hot-air drying (HAD), vacuum drying (VD), infrared drying (IRD), and freeze-drying (FD).

## Data Availability

The datasets produced in this study are available upon request from the corresponding author.

## Supplementary Materials

The following supporting information can be downloaded at: https://www.mdpi.com/article/10.3390/antiox13121554/s1, Figure S1: Correlation analysis visualized by heatmap between the bioactive compounds and antioxidant, antiproliferative and antidiabetic potential of dried blueberries. Dark red and blue report the degree of significantly positive and negative correlations, respectively.
