# Supplementary figures and images for: Drying: A Practical Technology for Blueberries (Vaccinium corymbosum L.)—Processes and their Effects on Selected Health-Promoting Properties
Source: Antioxidants (Basel). 2024 Dec 18;13(12):1554. doi: 10.3390/antiox13121554 (PMC11673246; doi:10.3390/antiox13121554)

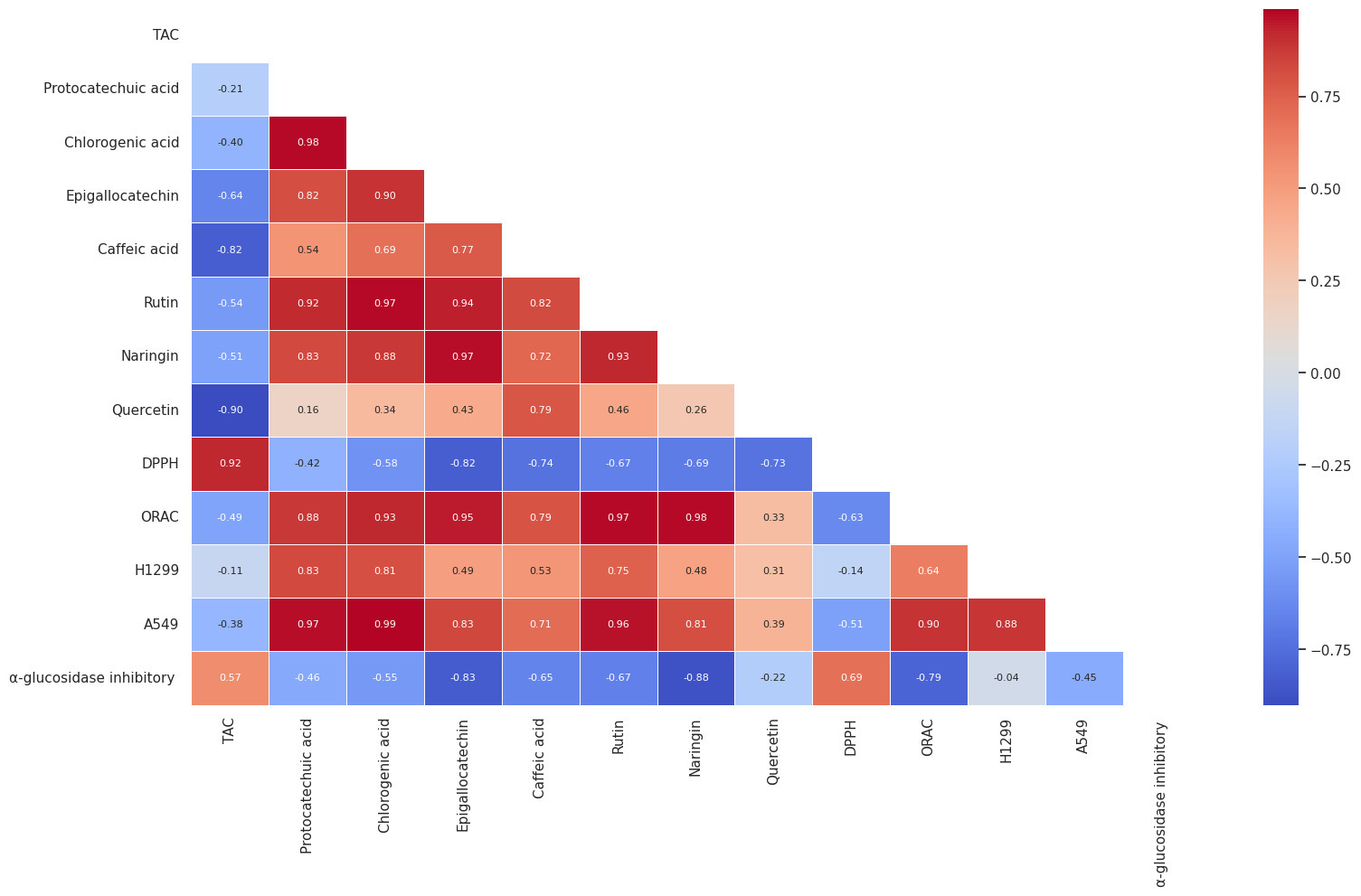

Supplement: Supplementary file 1 [file antioxidants-13-01554-s001.zip › antioxidants-3342137-supplementary.jpg]
